# Detection of *Ichthyophthirius multifiliis* (Ichthyophthiriidae) in two wild amphibian species

**DOI:** 10.3389/fvets.2025.1682567

**Published:** 2025-12-01

**Authors:** Srisupaph Poonlaphdecha, Albert Martínez-Silvestre, Norma Collado Conde, Joan Budó Ricart, Nannaphat Suwannarat, Alexis Ribas

**Affiliations:** 1 Parasitology Section, Faculty of Pharmacy and Food Science, Department of Biology, Healthcare and Environment, University of Barcelona, Barcelona, Spain; 2 Institut de Recerca de la Biodiversitat (IRBio), Universitat de Barcelona, Barcelona, Spain; 3 CRARC-Catalonian Reptiles and Amphibians Rescue Center, Masquefa, Spain; 4 CRT-Centre Reproducció Tortugues, Garriguella, Spain; 5 Program in Fishery Science and Aquatic Resources, Department of Agricultural Technology, King Mongkut’s Institute of Technology Ladkrabang, Prince of Chumphon Campus, Chumphon, Thailand

**Keywords:** *Ichthyophthirius*, *Salamandra*, drought, amphibian, Spain, aquatic ecology, disease

## Abstract

Emerging infectious diseases are one of the main threats to global amphibian populations. Frogs and salamanders are already affected by various pathogens, including ranaviruses, *Batrachochytrium dendrobatidis*, *B. salamandrivorans*, and helminths. Here, we report the first confirmed cases of *Ichthyophthirius multifiliis*, a ciliate parasite traditionally considered fish-specific, infecting wild amphibian larvae in a natural setting. As part of a passive surveillance program in Catalonia, five dead amphibians (four *S. salamandra* larvae and one *Rana temporaria* post-metamorph) were collected from a freshwater spring and examined for common pathogens. All individuals tested negative for chytrid fungi and ranaviruses by qPCR. However, histological examination of gill tissue revealed the presence of *I. multifiliis* trophonts in three of the salamander metamorphs and in the frog post-metamorph. In addition, larvae of forty-two amphibians from other localities in Catalonia tested negative. The observed trophonts matched morphological features previously described in teleost infections, including their characteristic macronucleus and surface ciliation. Our results further validate the previous molecular detections and experimental evidence suggesting the parasite’s potential for cross-taxon infection, raising concerns about the overlooked potential prevalence of *I. multifiliis* in wild amphibians. Given the increasing impact of climate change and habitat alteration on global freshwater ecosystems, this study highlights the importance of including *I. multifiliis* in amphibian disease monitoring programs.

## 1 Introduction

Globally, and in Spain, amphibian populations are threatened by a variety of emerging infectious agents such as bacteria (red-leg syndrome), ranaviruses, chytrid fungi (*Batrachochytrium dendrobatidis* and *B. salamandrivorans*), and parasites (helminths) (1–6). *Ichthyophthirius multifiliis*, which causes Ichthyopthiriasis (also referred to as Ich or white spot disease), is a ciliated protozoan parasite that primarily infects the skin and gills of freshwater teleost fish (7). It is considered one of the most economically significant parasites in aquaculture due to its rapid transmission, high virulence, and potential for substantial mortality, especially in high-density rearing environments (7). Disease sequelae occur in infected fish that are environmentally stressed, typically in association with warmer water temperatures (7). The parasite has a direct life cycle consisting of three main stages: the tomont, a reproductive stage that encysts on environmental substrates; the theront, a free-swimming infective stage that actively seeks new hosts; and the parasitic trophont stage, that embeds itself in the epithelium of the fish host, usually in the skin and gills (8). The trophont stage of the parasite can be macroscopically visible as white spots while feeding and growing on infected fish tissue and microscopically identified by its large, horseshoe-shaped macronucleus.

Several protozoan diseases, involving ciliates, flagellates, or amoeboid protists inhabiting the skin, gills, or gastrointestinal tract, are well documented in freshwater amphibian species, including the fire salamander (*Salamandra salamandra*) and the European common frog (*Rana temporaria*) (4, 5, 9, 10). Although *I. multifiliis* is a ubiquitous parasite of most freshwater fish species, it is not considered a natural host of freshwater amphibian species (11). Only two previous studies have reported amphibian associations with *I. multifiliis*: a presumptive molecular detection of the parasite was found in asymptomatic Natterjack toads (*Bufo calamita*) and striped marsh frog (*Limnodynastes peronii*) tadpoles were susceptible by experimental exposure (11, 12).

The present study is based on a passive surveillance approach of assessing amphibian health in Catalonia, whereby herpetologists, park rangers, and members of the environmental and rural police force collect dead or moribund amphibians encountered in the field. Specimens suspected to have succumbed to pathogenic infections are subsequently transferred to the appropriate centers and laboratories for a thorough diagnostic investigation.

In this case study, five dead amphibians (four fire salamander metamorphs and a single common frog tadpole) were collected from a natural spring fountain in Catalonia and examined for *I. multifiliis*. Our efforts to monitor the potential emergence of this fish parasite in new amphibian host species enhances our understanding of pathogen host shifts and contributes to the overall conservation efforts aimed at preserving wild amphibian populations.

## 2 Materials and methods

In July 2024, three dead larvae of the fire salamander (*S. salamandra*) were found in a spring and collected by the environmental and rural police. Later, in October 2024, the site was revisited, and one additional dead larva of *S. salamandra* and one larva of *R. temporaria* were collected for laboratory analysis.

**Figure 1:**
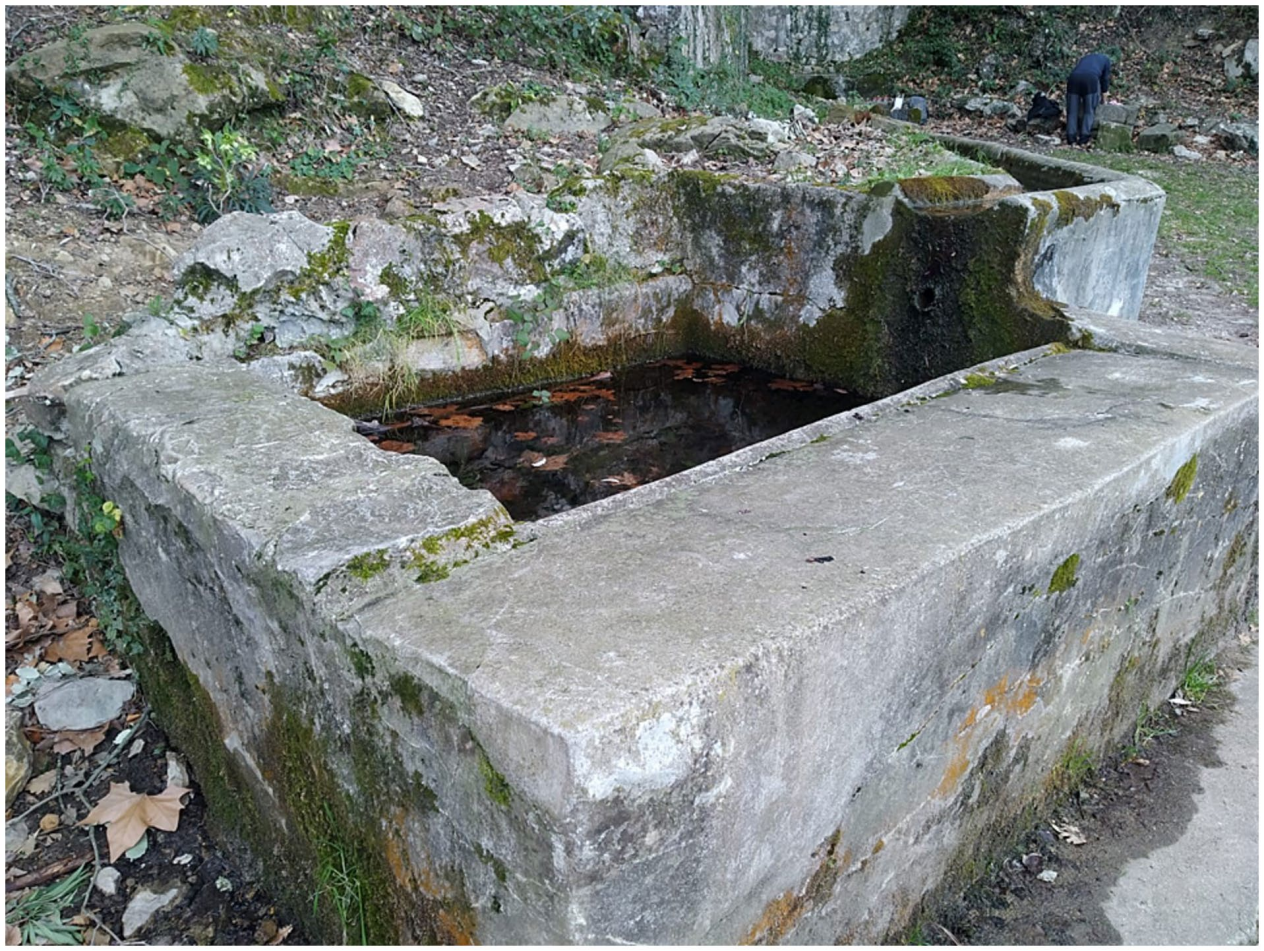
Natural spring fountain in Catalonia (Spain) from which dead amphibian larvae were collected in the summer of 2024.

The spring is located in the municipality of Beuda (Garrotxa county) (42.25797°N, 2.737534°E), a few meters from the border of the Alt Empordà county, at an altitude of 749 m above sea level (Figure 1). The fountain with flowing spring water, known as “Can Falgars,” is situated 200 m from a centuries-old farmhouse. The surrounding forest is dense and mature, predominantly composed of holly oak (*Quercus ilex*), with some downy oaks (*Quercus pubescens*). Along the spring and the stream that flows from it, the field maple (*Acer campestre*) and holly (*Ilex aquifolium*) grow naturally. There are also some silver firs (*Abies alba*) and plane trees (*Platanus* × *hispanica*) planted long ago. The soil of this mountain ridge, oriented perpendicular to the main axis of the Pyrenees, is composed of limestone. From the spring, where water naturally emerges from a wall of rocks, a trough of 25 cm wide and 15 cm deep emerges, which runs for 17 m leading the water to a small basin measuring 1 × 1.40 m, with a depth of about 0.70 m. From there, the water overflows and forms a small natural stream flowing down the mountainside. The site is adjacent to a trail frequently used by hikers and mountain bikers, where the presence of domestic dogs is possible. Additionally, a herd of cattle grazes in the surrounding area throughout the year. No fish were observed in the nearby surface-exposed flowing spring water or at the collection site (e.g., the fountain).

All collected specimens were immediately transported under refrigeration to the laboratory and screened for the presence of pathogens (*B. dendrobatidis*, *B*. *salamandrivorans,* and ranaviruses). DNA was extracted from the swab samples (a single swab covering both ventral and dorsal skin and gills) using PrepMan Ultra, following the protocol of reference (13). A real-time TaqMan PCR assay was conducted on a MyGo Pro machine, using the protocol of reference (14) for the detection and quantification of *B. dendrobatidis*, *B*. *salamandrivorans*, and that of reference (15) for ranaviruses. All samples were run against negative and positive controls with known genomic equivalent concentrations of zoospores/virions, ranging from 0.1 to 10,000,000 in log10 increments. A sample was considered positive if the infection load was equal to or greater than that of the lowest positive control and the amplification curves showed clear sigmoidal shapes.

No wet mount of a gill clip and/or skin scrape was performed after specimen receipt at the lab, and intact specimens were immediately preserved in formalin after swabbing. Histological sections of the gills were prepared by longitudinally sectioning the full larvae. The larvae were first fixed to preserve cellular structures, dehydrated, cleared, and paraffin embedded to facilitate sectioning. Four-micrometer sections were cut and mounted on slides before sequential staining with hematoxylin and eosin (H&E). Histological slides of *Ichthyophthirius multifiliis* from fish maintained in the Section of Parasitology at the University of Barcelona were used as references to compare with the positive samples of the present study. The prepared slides were observed under a microscope (MOTIC BA-210 LED, trinocular head), connected to a PC camera (MOTICAM S12), with software (Motic Images Plus 3.1) for image capture. The total number of *I. multifiliis* present in the gill sections of each specimen was counted under the microscope (×100 magnification) for estimation of parasite intensity; the number of fields observed per specimen ranged between two and five.

In addition, our archival collection of amphibian larvae collected between 2018 and 2025 (*n* = 42) was rechecked using histological techniques for the presence of *Ichthyophthirius multifiliis.* This included *Salamandra salamandra* (*n* = 22), *Triturus marmoratus* (*n* = 12), *Lissotriton helveticus* (*n* = 5), *Calotriton asper* (*n* = 2), and *Pleurodeles waltl* (*n* = 1) from various localities across the provinces of Barcelona, Tarragona, and Girona.

## 3 Results

**Figure 2:**
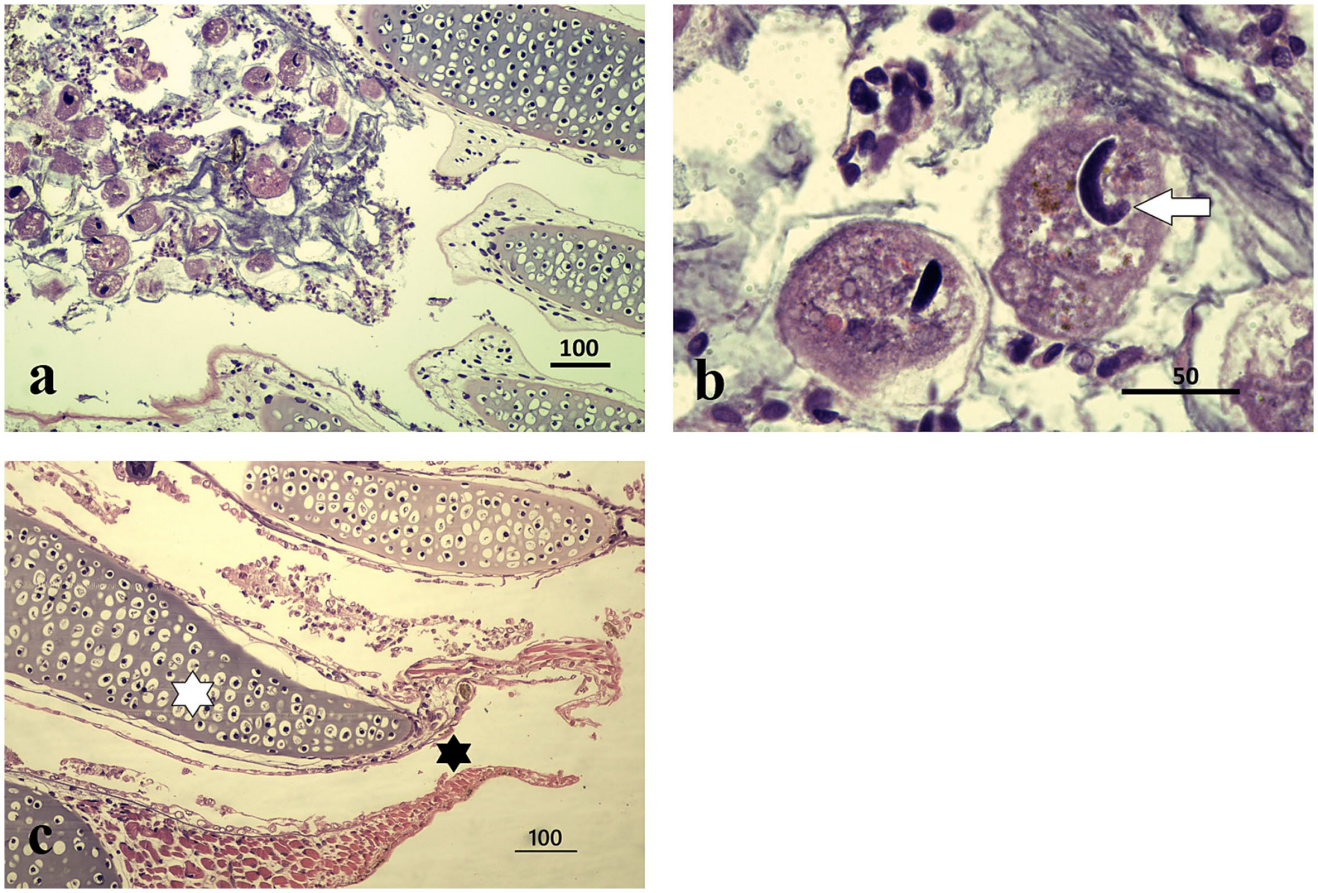
Histopathological features of *I. multifiliis* infection in *S. salamandra*. **(a)** Parasite presence in gill tissue, distributed within epithelial layers; protozoa are attached to the external cell layers without affecting the cartilaginous structure. Magnification ×100, scale bar 100 μm. **(b)** Close-up of a trophont, illustrating characteristic morphology and interaction with host tissue. Magnification ×1000, scale bar 50 μm. **(c)** Healthy gills of an individual from other locality, at the center of each gill filament lies a cartilaginous core providing structural support (white asterisk). Surrounding this core is a thin layer of epithelial cells specialized for gas exchange (black asterisk). This epithelium is primarily made up of pavement cells, ciliated cells, and mitochondria-rich cells; magnification ×100, scale bar 100 μm.

**Figure 3:**
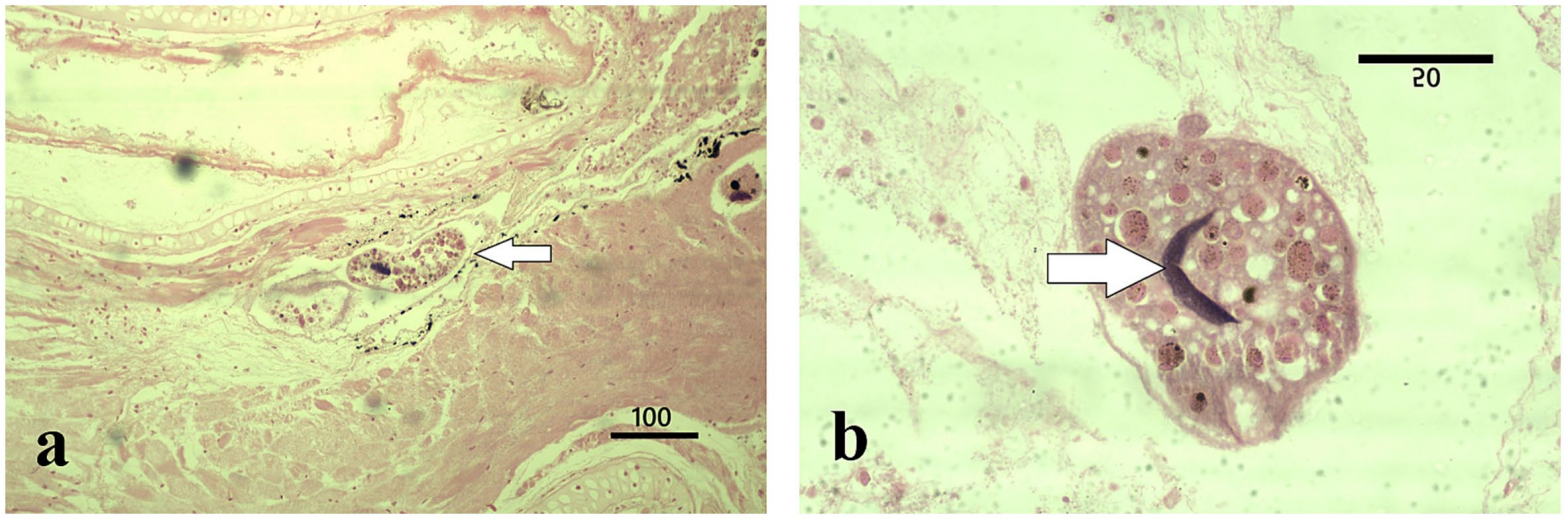
Histopathological features of *I. multifiliis* infection in *R. temporaria*. **(a)** Parasite presence in gill tissue (arrow); the cellular layers adjacent to the parasites are altered by a state of incipient autolysis. Magnification ×100, scale bar 100 μm. **(b)** Close-up of a trophont, illustrating characteristic morphology with a C-shaped nucleus (arrow) and interaction with host tissue. Magnification ×1000, scale bar 50 μm.

Detailed external examination in the laboratory revealed no white spots, hemorrhages, or any other clinical signs on the skin or gills of the collected specimens (*n* = 5 larvae; four salamander metamorphs and one frog post-metamorph). The qPCR testing for ranaviruses, *Batrachochytrium dendrobatidis,* and *Batrachochytrium salamandrivorans* was negative in all five samples. However, two of the three salamanders collected in July 2024 and the two specimens (one salamander and one frog) from October 2024 collection were positive for *Ichthyophthirius multifiliis* (Figures 2a,b, 3). All five larval amphibian species (*n* = 42 individual specimen tissues), including the 22 *S. salamandra* specimens, from our archival histological collection, that were rescreened for *I. multifiliis* showed no evidence of ciliated parasites (Figure 2c).

Microscopic examination of the stained gill sections revealed the presence of *Ichthyophthirius multifiliis* trophonts embedded within the epithelial layers, consistent with infections typically observed in teleost fish. The overall mean number of trophonts in salamander gills was twelve (range 1–62), whereas one trophont was observed in the frog gill tissue. The trophonts exhibited the characteristic morphology of *I. multifiliis*, appearing spherical to ovoid in shape, with short cilia covering the entire surface. Each trophont possessed a single, round to oval macronucleus and a single, smaller micronucleus, as described by the reference (16) (Figures 2a,b, 3). Histologically, the trophonts were a large, spherical to amoeboid ciliates, surrounded by a thickened membrane, and located within the epidermal or gill epithelial layers of the host. They possessed a horseshoe-shaped macronucleus and an inconspicuous cytostome. The cytoplasm contained abundant extrusive organelles, notably mucocysts (crystalline, oval-shaped, arranged as rosettes beneath the membrane), and rod-shaped toxicysts. The tissue surrounding the trophonts exhibited a chronic inflammatory response characterized by a predominance of lymphocytes. In some cases, there was a discrete accumulation of fibrin in the periparasitic area, further indicating an ongoing tissue response to the parasite (Figure 2b). Frog tadpole gill tissue clearly demonstrated clear trophont infiltration (Figure 3a), with trophont morphology identical to that observed in salamander tissue sections (Figure 3b).

## 4 Discussion

In this case study, we report the first confirmed occurrence of trophonts of *Ichthyophthirius multifiliis* infecting wild-caught amphibians, specifically the larvae of the fire salamander (*Salamandra salamandra*) and the European common frog (*Rana temporaria*). Our detection of the parasite provides further evidence that amphibians are indeed a permissive hosts of *I. multifiliis* and that infections can occur in larval salamanders and frogs under natural settings. Gleeson (11) previously demonstrated experimentally that striped marsh frog (*Limnodynastes peronii*) tadpoles developed clinical ichthyophthiriasis at high parasite loads. In the wild, *I. multifiliis* DNA has also been detected in toe-clip samples from the toad *Bufo calamita* in Portugal, though this finding does not confirm active infection. Such detections may instead reflect environmental contamination or passive contact, given that the tomont stage creates a sticky capsule capable of adhering to various substrates (12). This finding suggests a broader host range for *I. multifiliis* than previously recognized and raises important ecological and epidemiological questions regarding amphibian susceptibility and their role in parasite transmission within shared aquatic habitats. Amphibian larvae may become exposed to *I. multifiliis* through ecological overlap with infected fish in freshwater habitats. Although white spot disease is generally associated with cultured fishes, recent outbreaks have been reported in wild fish (17, 18), especially under conditions of environmental stress or compromised immune function. Our findings expand the list of potential emerging pathogens in the two amphibians studied. In salamanders, *Batrachochytrium salamandrivorans* is recognized as a rapidly emerging fungal pathogen (19), and an unidentified etiological agent, likely a protist, has been reported in Italy (20). In *Rana temporaria*, a novel tamanavirus (Flaviviridae) was recently described in the United Kingdom (21), and the presence of ranid herpesvirus in this species has also been documented (22, 23).

In addition, encysted tomonts could potentially colonize new water bodies through accidental transport by animals or humans. Trophonts of *I. multifiliis* embed within the epithelial tissues of fish, primarily adhering through mucosal contact and exploiting epithelial damage. While the parasite is considered fish-specific, these attachment mechanisms may not be entirely exclusive to fish. Gills of amphibian larvae share structural features with fish epithelia, such as mucus secretion, epithelial cilia, and keratinized layers, that might allow for temporary or opportunistic adhesion. Although the full life cycle of *I. multifiliis* may not be completed in amphibians or reptiles, temporary colonization or pseudo-infection cannot be ruled out, particularly under stress-induced physiological changes.

Environmental conditions likely facilitated the emergence of this infection. From 2021 to mid-2024, Catalonia experienced its most severe drought on record, leading to critically low water levels, dam depletion, and widespread water restrictions (24). Concurrent climate warming has further exacerbated ecological pressures on amphibian populations, increasing concerns about species persistence under rising temperatures (25–27). Such environmental disturbances alter aquatic ecosystems and may favor protozoan proliferation through elevated water temperatures and stagnant flows. These conditions have previously been linked to *I. multifiliis* outbreaks in fish, as demonstrated by (17) in redtail barbs during drought in a Catalan stream.

In this study, we report for the first time the presence of *I. multifiliis* in the gills of wild-caught amphibians, specifically in the fire salamander (*Salamandra salamandra*) and the European common frog (*Rana temporaria*). This finding confirms that *I. multifiliis* does not exhibit strict host specificity, as experimental studies have suggested, and points to broader ecological plasticity. Although direct causation between parasite presence and host mortality could not be established, the infection may contribute to overall stress and disease susceptibility. Prior to this study, surveys for the presence of *I. multifiliis* in amphibians in Catalonia were not conducted systematically but only occasionally, with the few individuals examined testing negative. The lack of comprehensive monitoring may partly explain why this ciliate had not been previously reported in wild amphibian populations. A systematic study of amphibian larvae gills would help establish its presence. Other fish-specific pathogens have also been reported to infect amphibians. For example, a rhabdovirus typically associated with carp species (*Carpio sprivivirus*) has caused mortality in adult Chinese firebelly newts (*Cynops orientalis*) (28). Experimental infections further demonstrated lethal effects in two North American amphibians: metamorphs of the western long-toed salamander (*Ambystoma macrodactylum*) and tadpoles of the Pacific treefrog (*Pseudacris regilla*) (29).

The detection of *I. multifiliis* in non-fish vertebrates underscores the need to reevaluate the parasite’s ecological dynamics. Cross-species infection studies are warranted to examine the potential for transmission in freshwater environments where fish, amphibians, and reptiles coexist. Given the vulnerability of amphibians to multiple environmental stressors, including pathogens, climate change, and habitat alteration, our findings highlight the importance of including protozoan surveillance in amphibian conservation and health monitoring programs.

## Data Availability

The original contributions presented in the study are included in the article/supplementary material, further inquiries can be directed to the corresponding author.
